# Prediction of incisional hernia after kidney transplantation: analysis of wound closure technique and risk factors

**DOI:** 10.1007/s10029-025-03452-2

**Published:** 2025-08-19

**Authors:** Kristoffer Huitfeldt Sola, Torkel Brismar, Tomas Lorant, Ulf Fränneby, Oskar Larsson, Helena Genberg

**Affiliations:** 1 Division of Radiology, CLINTEC, Karolinska Institutet, Stockholm, Sweden; 2 Department of Radiology, Karolinska University Hospital, Stockholm, Sweden; 3 Department of Surgical Sciences, Section of Transplantation Surgery, Uppsala University, Uppsala, Sweden; 4 Division of Surgery, Karolinska Institutet. Department of Surgery, CLINTEC, Karolinska University Hospital, Stockholm, Sweden; 5 Division of Transplantation Surgery, CLINTEC, Karolinska Institutet, Stockholm, Sweden; 6 Department of Transplantation Surgery, Karolinska University Hospital, Stockholm, Sweden

**Keywords:** Incisional hernia, Kidney transplantation, Wound closure technique, Risk factors

## Abstract

**Purpose:**

Incisional hernia (IH) is a common complication after kidney transplantation, impacting morbidity and quality of life. This retrospective study aimed to identify IH risk factors and develop a predictive model.

**Methods:**

We retrospectively analysed 667 adult kidney transplant recipients (2010–2017) from two transplant centres. Medical records were screened for symptoms of abdominal wall impairment, postoperative CT scans assessed, and factors associated with IH analysed. Using the Penn Hernia Calculator, hernia probability was calculated. Adult kidney recipients transplanted 2018–2019 in Region Stockholm served as verification cohort. In a subgroup with preoperative CT scans after progression to stage 5 chronic kidney disease, muscle quality was assessed. A wound closure technique using self-locking knots, two-layer parietal running suture, and a suture-to-wound length ratio ≥ 4:1 was termed “modified Israelsson.”

**Results:**

Logistic regression identified age, BMI, renal replacement therapy duration, and wound closure technique as independent IH risk factors (pseudo R² = 0.15). The “modified Israelsson method” reduced IH odds by 83% (OR = 0.17). Sarcopenia and myosteatosis were not significant predictors. In the verification cohort, the model had 76% sensitivity for high-risk patients (≥ 10% predicted IH risk), outperforming the Penn Hernia Calculator.

**Conclusion:**

Wound closure technique is the strongest modifiable predictor of symptomatic IH identified in this cohort. The “modified Israelsson method” is a straightforward technique that shows strong promise for reducing incisional hernia (IH) rates and appears highly implementable. Our findings also underscore the value of developing specific predictive models for kidney transplant recipients, as generic tools may not capture crucial intraoperative factors.

## Introduction

Incisional hernia (IH) after kidney transplantation is a common complication that significantly reduces quality of life [1]. Known risk factors for IH include age, female gender, history of smoking, obesity, and duration of dialysis dependence [2, 3]. Patients with IH often suffer from pain, discomfort, and limitations in daily activities, leading to poorer health-related quality of life scores, particularly in physical functioning, bodily pain, and vitality. Many also report dissatisfaction with their body image and reduced sexual activity compared to those without IH [1].

Kidney transplantation is usually performed via a low lateral abdominal incision, typically referred to as the Gibson incision. To our knowledge, no study has assessed the influence of the wound closure technique on the risk of IH development after kidney transplantation. In 2013 Israelsson et al. proposed a technique for wound closure of midline incisions which involves certain significant key points, the most important being self-locking knots, running suture and a suture to wound length ratio of > 4:1 [4]. However, the efficacy of this method has not been evaluated for lateral incisions.

Incisional hernia is also a common complication after midline laparotomy, leading to significant morbidity and increased healthcare costs. The short-stitch technique has been shown to reduce hernia rates, with a trial indicating fewer burst abdomen events compared to the standard long-stitch method [5]. Additionally, the HART trial, compared the Hughes abdominal closure method with standard mass closure in 802 patients undergoing colorectal cancer surgery. While there was no significant difference in IH occurrence at one year between the two techniques, the trial highlighted the limitations of clinical examination compared to computed tomography in detecting hernias [6].

Recently, the Penn Hernia Risk Calculator was introduced to predict the risk of IH following a range of intraabdominal procedures, including kidney transplantation [7]. Factors such as age, race, BMI, smoking history, drug abuse and medical conditions are used in the calculations, while intraoperative parameters such as the wound closure technique are omitted. Yet, it is well-established that wound complications following midline incision are strongly related to wound closure technique [4]. The precision of prediction tools for IH using patient factors only may therefore be questionable.

Sarcopenia is a condition characterized by the progressive loss of muscle strength and has been put forward as a significant factor in the development of incisional hernia after surgery [8]. The loss of muscle quality and muscle quantity in sarcopenia could lead to weakness of the abdominal wall, making it more vulnerable to IH formation. In addition, sarcopenia is associated with an increased risk of multiple postoperative complications, including infection, which could further compromise the integrity of the abdominal wall [9]. A prior study on midline incisions found no correlation between sarcopenia and incisional hernia development [10]. However, it is unclear whether this also applies to incisions in the lateral abdominal wall area, often involving transection of muscle and not only connective tissue.

There are numerous ways of estimating whole body skeletal muscle mass but the gold standard is a computed tomography (CT) image taken at the third lumbar (L3) level [11]. The area of the skeletal muscle at the L3 level is adjusted by the squared height of the patient, so-called skeletal muscle index (SMI), and the resulting value is used to diagnose sarcopenia applying pre-existing cut-offs. However, these cut-off values vary widely between studies and there is currently no consensus [12–14]. By adjusting the thresholds at segmentation, one can also measure the area of low attenuation muscle or “fatty-muscle” by similar methodology [15].

With the aim of developing an IH prediction model, in this study an analysis of patient-related risk factors as well as intraoperative and postoperative factors associated with IH in kidney transplantation was undertaken, including a subgroup analysis on the impact of sarcopenia.

## Methods

All adult (> 18 years) kidney recipients residing in Region Stockholm and Region Gotland, receiving a kidney transplant between January 2010 and December 2017 at Karolinska University Hospital Stockholm, and adults, residing in Region Uppsala, transplanted between January 2010 and December 2017 at Uppsala University Hospital were screened retrospectively. Subjects with a previous ipsilateral kidney transplantation, a multiorgan transplant, and those without a Swedish social security number were considered ineligible for the study. Kidney recipients who died within 3 months of transplantation were also excluded. A verification cohort was created from all adult (> 18 years) kidney recipients in Region Stockholm and Region Gotland transplanted between January 2018 and December 2019 using the same inclusion criteria.

In the subgroup analysis of sarcopenia, eligible patients were those with a preoperative CT scan within 36 months of transplantation and after progression to stage 5 chronic kidney disease (CKD-5). The CT images of the L3 level were exported and the area of muscle measured using preestablished cut-offs for both skeletal muscle (−29 to + 150 Hounsfield units) and fatty muscle (−29 to + 30 Hounsfield units). The resulting value was divided by height squared (m^2^) to provide the SMI and fatty skeletal muscle index (FMI). Only patients transplanted in Region Stockholm and Region Gotland were considered for the subgroup analyses owing to restrictions in the ethical approval. All measurements were performed in Image J (National Institute of Health) [16].

Data were retrieved from the electronic medical records, Swedish renal registry, Scandiatransplant registry and the hospitals’ internal picture archiving systems. Medical records were screened for reported symptoms of abdominal wall impairment at the site of surgery and clinical parameters relevant to the study were obtained. Postoperative abdominal CT scans were retrieved and were all multidisciplinary reassessed by the researchers applying the European Hernia Society’s definition of a hernia. The health administrative database using the ICD-10 coding system was used for data verification. Procedures that occurred between midnight and 7 am were considered night-time transplantations. Kidney donor profile index (KDPI) and living kidney donor profile index (LKDPI), which quantify the risk of graft failure after kidney transplantation, were calculated using standardized online calculators [7]. The last follow-up date was April 1, 2024, i.e. at least 5 years of follow-up.

**Fig. 1:**
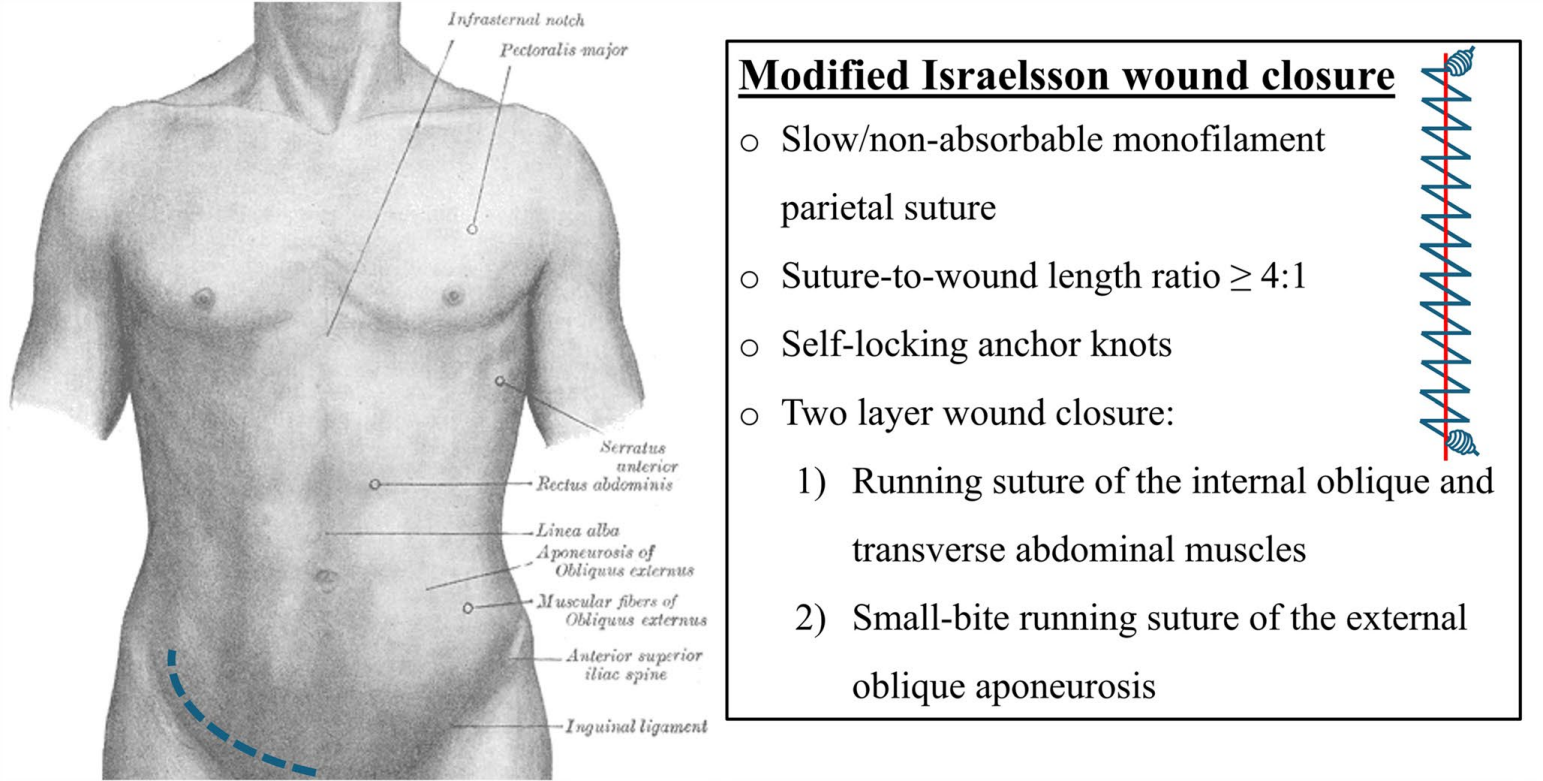
Schematic illustration of the modified Israelsson technique. The method includes a two-layer parietal closure using slow/non-absorbable monofilament sutures, self-locking anchor knots, and a suture-to-wound length ratio ≥ 4:1. The deep parietal layer involves a running suture of the internal oblique and transverse muscles; the superficial layer closes the external oblique aponeurosis with a small-bite running suture. The anatomical image of the abdominal surface is adapted from *Gray’s Anatomy*, 20th edition (1918, public domain)

Information about wound closure technique was retrieved from the surgery report and through a questionnaire to the surgeons. A wound closure technique was defined as “modified Israelsson” if it included the following: slow or non-absorbable monofilament parietal sutures, a suture-to-wound length ratio ≥ 4:1, self-locking anchor knots, and a two-layer parietal closure, with a running suture of the internal oblique and transverse muscles, and a small-bite running suture of the external oblique aponeurosis (Fig. 1). If the technique could not be definitively determined after these steps, the case was not classified as modified Israelsson and was treated as a standard wound closure.

Statistical analyses were conducted using R Core Team (2013) (R Foundation for Statistical Computing, Vienna, Austria), with a p-value of < 0.05 considered statistically significant. Initially, univariate logistic regression analyses were performed to identify associations between individual risk factors and the development of IH. A stepwise multivariate logistic regression was then used to refine these findings by including only variables that showed significance in the univariate analysis. Chi-squared tests and Wilcoxon rank sum tests were applied in subgroup analyses to compare categorical and continuous patient characteristics, respectively.

## Results

**Fig. 2:**
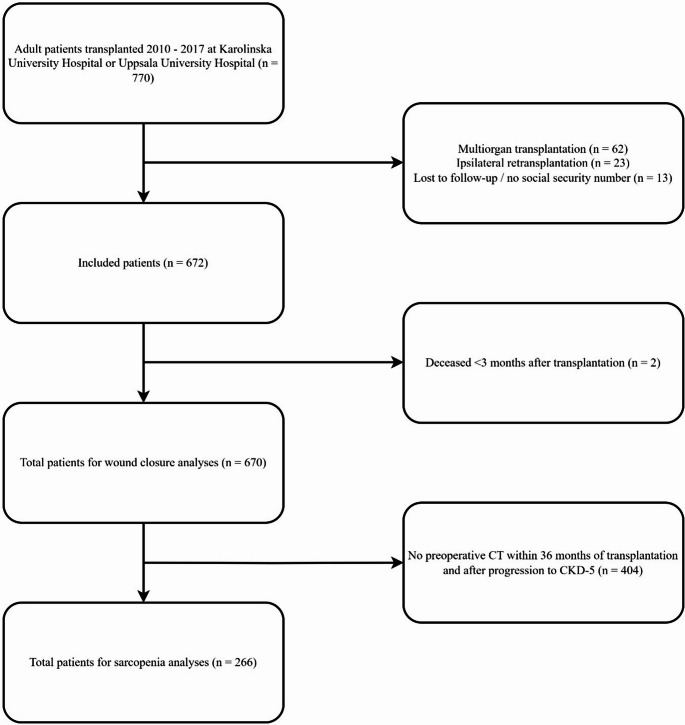
Flow chart of included and excluded study participants showing both total number of patients for wound closure analyses, and total number of patients for the sarcopenia sub-analysis

Out of 770 adults transplanted between 2010 and 2017, 670 met the inclusion criteria for the study (87%) and 266 (40%) met the criteria for the subgroup analysis on sarcopenia (Fig. 2). The median age of the whole study population was 53.9 years, median BMI, 25.2 kg/m^2^, 64% were male, and 85% received a standard triple immunosuppressive protocol including tacrolimus, mycophenolate and prednisolone (Table 1.). Postoperative abdominal CT scans were available for 51% of study participants at a median of 37 months postoperatively. Symptomatic IH was reported in 88 (13%) of the 670 study participants.

**Table 1:** Study population characteristics. Values in parentheses are percentages unless indicated otherwise. Parameters with ^1^ are given with median (interquartile range). Statistical analyses used are Wilcoxon rank sum test for numerical data, and Chi-squared tests for categorical data

Pre and intraoperative factors			Without symptomatic IH (*n* = 582)	With symptomatic IH (*n* = 88)		*P*-value	
Men (%)			381 (65)	49 (56)		= 0.114	
Age (per year)^1^			52.8 (41.3–62.2)	60.6 (54.4–64.1)		< 0.001	
Body mass index (kg/m^2^)^1^			25.0 (22.5–27.7)	26.7 (23.5–29.3)		= 0.016	
History of smoking						= 0.024	
Yes			285 (49)	55 (38)			
No			297 (51)	33 (63)			
Prior kidney transplantation						= 0.029	
First time kidney recipient			520 (89)	71 (81)			
Second time kidney recipient			62 (11)	17 (19)			
Duration of renal replacement therapy (years)^1^			1.2 (0.4–2.6)	2.0 (0.8–5.3)		= 0.001	
Type of renal replacement therapy						= 0.016	
Haemodialysis			295 (51)	59 (67)			
Peritoneal Dialysis			168 (29)	18 (20)			
Preemptive			119 (20)	11 (13)			
Donor Source						< 0.001	
Deceased donor			365 (63)	73 (83)			
Living donor			217 (37)	15 (17)			
KDPI/LKDPI-score^1^			49.6 (20.6–77.0)	64.0 (48.2–86.2)		< 0.001	
Polycystic kidney disease						= 0.448	
Yes			54 (9)	11 (13)			
No			528 (91)	77 (87)			
Diabetes						= 0.045	
Yes			93 (84)	15 (83)			
No			489 (16)	73 (17)			
Charlson Comorbidity Index^1^			3 (2–4)	4 (3–5)		< 0.001	
History of hernia						= 0.381	
Yes			118 (20)	22 (25)			
No			464 (80)	66 (75)			
CRP at transplantation			2 (0–4)	3 (1–8)		= 0.005	
Albumin at transplantation			35 (32–37)	34 (32–37)		= 0.438	
Haemoglobin at transplantation			116 (106 − 124)	118 (109–127)		= 0.125	
Wound closure technique						= 0.003	
Standard wound closure			488 (84)	85 (97)			
Modified Israelsson			94 (16)	3 (3)			
Intracutaneous suture							
Yes			81 (14)	8 (9)		= 0.282	
No			501 (86)	80 (91)			
Postoperative factors							
Re-exploration after transplantation						> 0.999	
Yes			115 (20)	18 (20)			
No			467 (80)	70 (80)			
Wound dehiscence						= 0.557	
Yes			16 (3)	4 (5)			
No			566 (97)	84 (95)			
Surgical site infection						= 0.001	
Yes			44 (8)	17 (19)			
No			538 (92)	71 (81)			
Anticoagulant treatment after transplantation						= 0.529	
Yes			368 (63)	52 (59)			
No			214 (37)	36 (41)			
Triple immunosuppressive protocol						> 0.999	
Yes			493 (85)	74 (84)			
No			89 (15)	14 (16)			
Mycophenolate treatment							
Yes			511 (88)	75 (85)		= 0.612	
No			71 (12)	13 (15)			
Azathioprine treatment						= 0.930	
Yes			54 (9)	9 (10)			
No			528 (91)	79 (90)			
mTOR treatment						> 0.999	
Yes			7 (1)	1 (1)			
No			575 (99)	87 (99)			
Low-dose corticosteroid regimen						= 0.862	
Yes			26 (5)	3 (3)			
No			556 (95)	85 (97)			
Transplant rejection treatment						= 0.864	
Yes			108 (19)	17 (19)			
No			474 (81)	71 (81)			
Graft failure						= 0.012	
Yes			47 (8)	15 (17)			
No			535 (92)	73 (83)			
Delayed graft function						= 0.022	
Yes			39 (7)	12 (14)			
No			543 (93)	76 (86)			
eGFR at 1 months			49.2 (34.9–62.8)	38.5 (27.3–55.6)		> 0.001	
eGFR at 12 months			50.6 (37.8–63.7)	46.1 (30.3–56.0)		= 0.002	

**Fig. 3:**
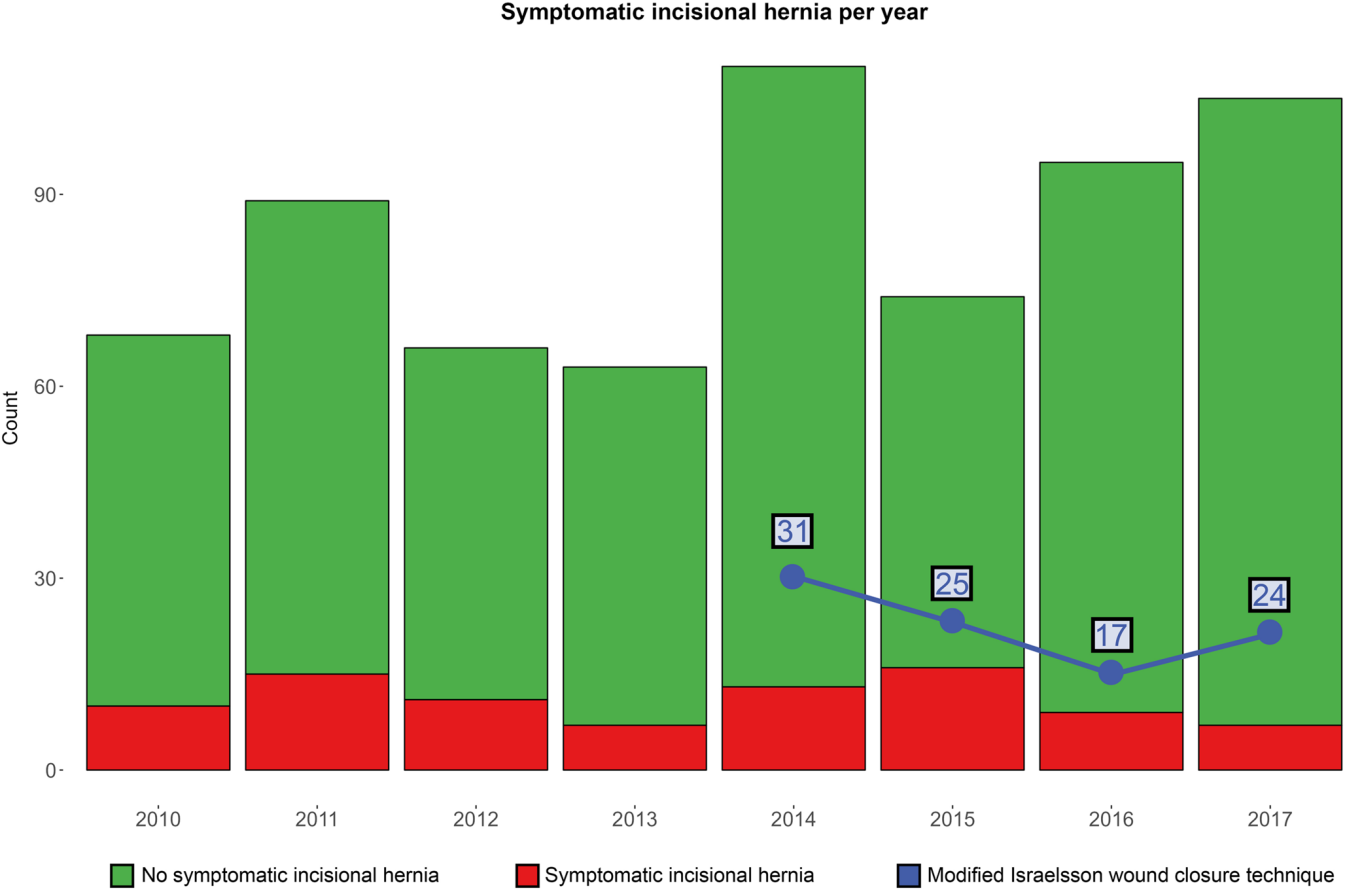
Number of symptomatic incisional hernias per year. The blue line corresponds to the number of surgeries performed according to the modified Isralesson wound closure technique per year

The number of symptomatic IH did not significantly differ between the years of transplantation (*p* =.101). Ninety-seven surgeries were performed according to the modified Israelsson technique, with these cases distributed between 2014 and 2017 (Fig. 3). Of 19 surgeons involved in the study, only one consistently performed the modified Israelsson wound closure technique.

Univariate logistic regression analysis on potential pre- and intraoperative risk factors of symptomatic IH showed significance for history of smoking, type of dialysis prior to transplantation, BMI at transplantation, KDPI/LKDPI-score, donor source, age at transplantation, duration of renal replacement therapy, prior kidney transplantation and wound closure technique (*p* <.05) (Table 1). Pre and intraoperative factors not significant in the univariate analysis were polycystic kidney disease, diabetes, hernia in the medical history, gender, C-reactive protein (CRP) at transplantation, albumin at transplantation, haemoglobin at transplantation, night-time kidney transplantation, intracutaneous suture, Charlson comorbidity index score and predicted hernia probability from the Penn Hernia Risk Calculator.

**Fig. 4:**
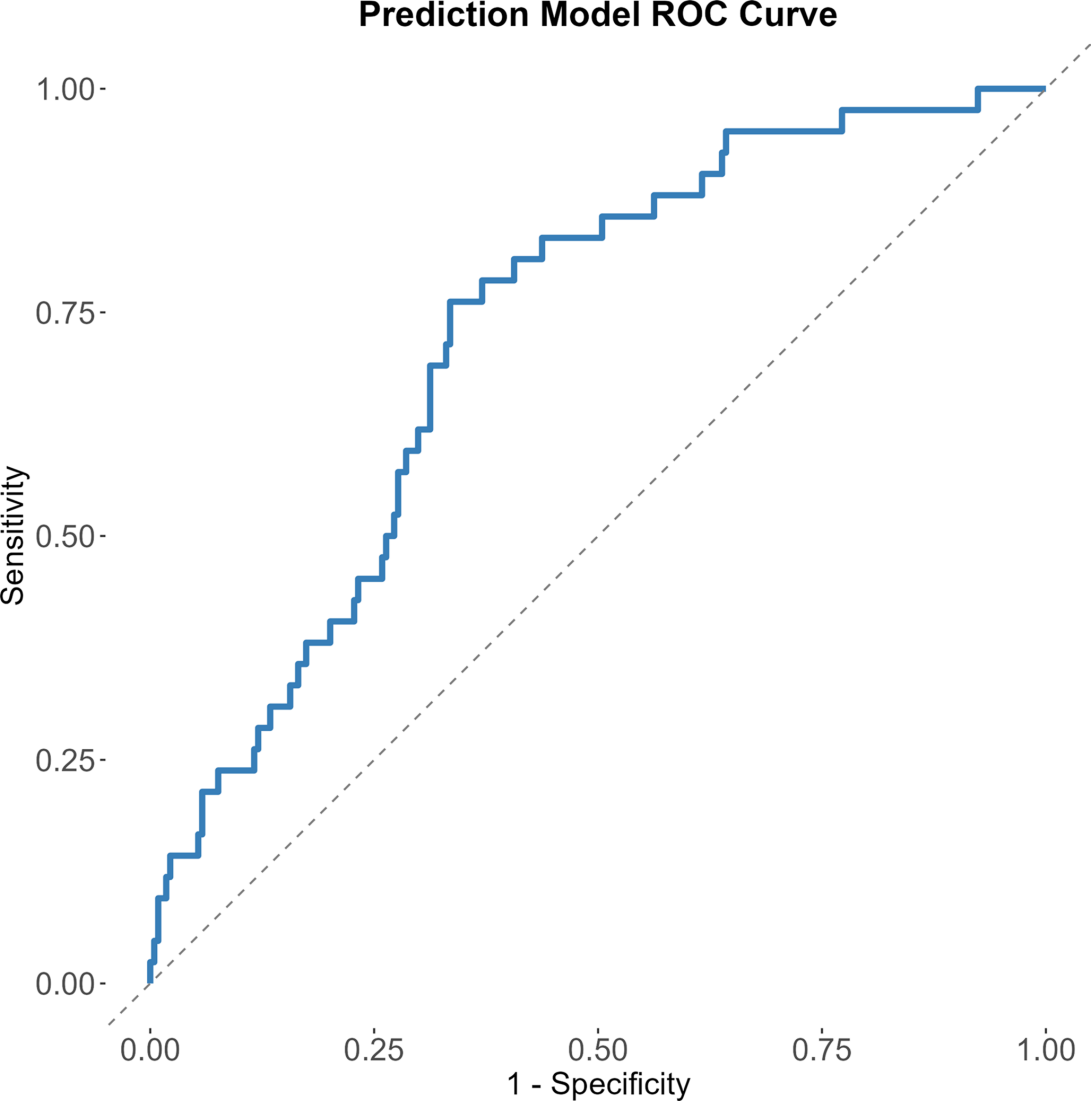
Receiver Operating Characteristic (ROC) curve for the final logistic regression model predicting symptomatic incisional hernia. The model includes age at transplantation, BMI at transplantation, duration of renal replacement therapy and wound closure technique. The ROC had an area under the curve (AUC) value of 0.72

In a stepwise multivariate logistic regression, prior kidney transplantation, type of dialysis before transplantation, smoking history, donor source and KDPI/LKDPI-score were not associated with IH. Increased age at transplantation, increased BMI at transplantation and longer duration of renal replacement therapy all increased the odds ratio of IH. Wound closure technique ad modum Israelsson however reduced the odds ratio (OR = 0.17). Thus, the final model with factors significant for IH development after stepwise multivariate logistic regression were: age at transplantation (years), BMI at transplantation, duration of renal replacement therapy (years) and wound closure technique (1, if the modified Israelsson surgical technique was used, 0 otherwise). The logistic regression had a pseudo R-squared of 0.15 according to the Nagelkerke method (Table 2). A receiver operating characteristic (ROC) curve for the final predictive model was created, with an Area Under the Curve (AUC) of 0.72 (Fig. 4).

**Table 2:** The final model with factors significant for IH development after Stepwise multivariate logistic regression

Multivariate logistic regression	OR (95% CI)	*P*-value
Increased age at transplantation (per year)	1.04 (1.02–1.06)	(*p* <.001)
Increased BMI at transplantation (per kg/m^2^)	1.07 (1.01–1.14)	(*p* =.023)
Duration of renal replacement therapy (per year)	1.08 (1.04–1.12)	(*p* <.001)
Modified Israelsson wound closure technique	0.17 (0.04–0.49)	(*p* =.004)

$$\begin{array}{lc}\mathrm{log}\left(\frac{\mathrm{p}}{1-p}\right)=-6.026+0.040\cdot\:\mathrm{Age}\\+0.069\cdot\:\mathrm{BMI}+0.080\cdot\:\mathrm{Renal}\:\mathrm{replacement}\:\mathrm{therapy}\\-1.744\cdot\:\mathrm{Israelsson}\end{array}$$

For postoperative factors, re-exploration after transplantation, wound dehiscence, anticoagulant therapy, mycophenolate treatment, azathioprine treatment, mTOR treatment, low-dose corticosteroid regimen and transplant rejection treatment did not correlate with IH development. However, the presence of a surgical site infection (OR = 3.0, *p* <.001), early graft failure (OR = 2.3, *p* =.008) and delayed graft function (OR = 2.2, *p* =.025) significantly increased the risk of IH. In contrast, high eGFR at 1 month after transplantation and 12 months after transplantation significantly correlated to reduced IH risk (OR = 0.9 per 1 ml/min/1.73, *p* <.001) and (OR = 0.9 per 1 ml/min/1.73, *p* =.002).

By excluding all patients with wound closure ad modum Israelsson (*n* = 97), factors significant for IH development in the univariate analysis were: history of smoking, type of dialysis prior to transplantation, BMI at transplantation, KDPI/LKDPI-score, donor source, age at transplantation, duration of renal replacement therapy and prior kidney transplantation. After a stepwise multivariate logistic regression analysis, factors significant in the final model were increased age at transplantation (*p* <.001), increased BMI at transplantation (*p* =.013) and the duration of renal replacement therapy (*p* <.001) (Nagelkerke = 0.12). Postoperatively, surgical site infection, delayed graft function and graft failure increased the risk of IH development in the univariate analysis.

Subjects with and without wound closure ad modum “modified Isralesson” showed no difference in patient characteristics except for donor source, type of renal replacement therapy before transplantation, albumin at transplantation and haemoglobin at transplantation (Table 3). Postoperatively there was a statistically significant difference in anticoagulant treatment, azathioprine treatment, mTOR treatment and low-dose corticosteroid regimen (Table 3).

**Table 3:** Comparison of patient characteristics and wound closure technique. Values in parentheses are percentages unless indicated otherwise. Parameters with ^1^ are given with median (i.q.r). Statistical analyses used are Wilcoxon rank sum test for numerical data, and Chi-squared tests for categorical data

Pre and intraoperative factors	Standard wound closure (*n* = 573)	Modified Israelsson (*n* = 97)	*P*-value
Men (%)	362 (63)	68 (70)	= 0.052
Age (per year)^1^	52 (40–62)	54 (42–63)	= 0.387
Body mass index (kg/m^2^)^1^	25.2 (22.6–28.0)	24.6 (22.7–27.7)	= 0.604
History of smoking			> 0.999
Yes	292 (51)	49 (51)	
No	281 (49)	48 (49)	
Prior kidney transplantation			= 0.317
First time kidney recipient	502 (88)	89 (92)	
Second time kidney recipient	71 (12)	8 (8)	
Duration of renal replacement therapy (years)^1^	1.4 (0.5–2.7)	1.1 (0–2.7)	= 0.23
Type of renal replacement therapy			= 0.034
Haemodialysis	313 (55)	41 (42)	
Peritoneal Dialysis	157 (27)	29 (30)	
Preemptive	103 (18)	27 (28)	
Donor Source			< 0.001
Deceased donor	179 (31)	53 (55)	
Living donor	394 (69)	44 (45)	
KDPI/LKDPI-score^1^	54 (24–79)	49 (17–77)	= 0.371
Polycystic kidney disease			
Yes	59 (10)	6 (6)	= 0.280
No	514 (90)	91 (94)	
Diabetes			= 0.092
Yes	98 (17)	10 (10)	
No	475 (83)	87 (90)	
Charlson Comorbidity Index^1^	3 (2–4)	3 (2–6)	= 0.178
History of hernia			= 0.950
Yes	119 (21)	21 (22)	
No	454 (79)	76 (78)	
CRP at transplantation	2 (0–5)	2 (0–4)	= 0.138
Albumin at transplantation	35 (32–37)	33 (30–37)	= 0.034
Haemoglobin at transplantation	117 (107–126)	112 (104–120)	= 0.006
Postoperative factors			
Symptomatic incisional hernia			= 0.003
Yes	85 (15)	3 (3)	
No	488 (85)	94 (97)	
Re-exploration after transplantation			= 0.732
Yes	112 (20)	21 (22)	
No	461 (80)	76 (78)	
Wound dehiscence			= 0.122
Yes	20 (3)	0 (0)	
No	553 (97)	97 (100)	
Surgical site infection			= 0.373
Yes	55 (10)	6 (6)	
No	518 (90)	91 (94)	
Anticoagulant treatment after transplantation			< 0.001
Yes	340 (59)	80 (82)	
No	233 (41)	17 (18)	
Triple immunosuppressive protocol			= 0.010
Yes	476 (83)	91 (94)	
No	97 (7)	6 (6)	
Mycophenolate treatment			= 0.225
Yes	497 (87)	89 (92)	
No	76 (13)	8 (8)	
Azathioprine treatment			= 0.004
Yes	62 (11)	1 (1)	
No	511 (89)	96 (99)	
mTOR treatment			= 0.001
Yes	3 (1)	5 (5)	
No	570 (99)	92 (95)	
Low-dose corticosteroid regimen			= 0.046
Yes	29 (5)	0 (0)	
No	544 (94)	97 (100)	
Transplant rejection treatment			= 0.978
Yes	107 (19)	18 (19)	
No	466 (81)	79 (81)	
Graft failure			= 0.188
Yes	57 (10)	5 (5)	
No	516 (90)	92 (95)	
Delayed graft function			= 0.324
Yes	46 (8)	5 (5)	
No	527 (92)	92 (95)	
eGFR at 1 months	48 (33–61)	49 (37–62)	= 0.472
eGFR at 12 months	50 (36–63)	51 (40–62)	= 0.531

Patients with “non-Israelsson wound closure technique” received kidneys from living donors in 31% of cases compared with 53% in the “modified Israelsson” group. In the univariate analysis the risk of IH was higher after deceased donor KT compared with living donor KT. However, the impact of donor source on the IH risk was not significant in the multivariate analysis.

Of 165 adults transplanted between January 2018 and December 2019, 158 were included in the verification cohort of whom 21 (13%) had symptomatic IH. If patients with a 10% risk of symptomatic IH, according to our model, were considered high-risk patients, 74 patients would have been flagged. Of those 16 were diagnosed with symptomatic IH. In comparison, using the Penn Hernia Risk calculator, two patients were flagged of whom 1 was diagnosed with symptomatic IH.

Similar subgroup analyses including sarcopenia were performed in the 266 patients with CT imaging available prior to transplantation. Univariate analysis was significant for history of smoking, age at transplantation, duration of renal replacement therapy and wound closure technique (*p* <.05). However, SMI and FMI were not significant risk factors of IH in the multivariate analysis. (Table 4).

**Table 4:** Subgroup analysis on patients with available CT imaging prior to transplantation. The final model was developed using a Stepwise multivariate logistic regression analysis, incorporating all parameters found to be significant in the univariate analysis, along with either the skeletal muscle index or the fatty muscle index

Subgroup analysis on SMI	OR (95% CI)	*P*-value
Skeletal muscle index (SMI)	0.98 (0.94–1.01)	(*p* =.209)
Increased age at transplantation (per year)	1.06 (1.02–1.10)	(*p* =.004)
Duration of renal replacement therapy (per year)	1.07 (1.01–1.13)	(*p* =.012)
History of smoking	2.71 (1.22–6.59)	(*p* =.019)
Modified Israelsson wound closure technique	0.21 (0.05–0.67)	(*p* =.019)
Subgroup analysis on FMI	OR (95% CI)	P-value
Fatty skeletal muscle index (FMI)	0.97 (0.91–1.02)	(*p* =.273)
Increased age at transplantation (per year)	1.07 (1.03–1.11)	(*p* =.002)
Duration of renal replacement therapy (per year)	1.07 (1.01–1.13)	(*p* =.012)
History of smoking	2.68 (1.21–6.51)	(*p* =.021)
Modified Israelsson wound closure technique	0.23 (0.05–0.72)	(*p* =.024)

## Discussion

To explore preventive measures, this study aimed to identify pre- and intraoperative risk factors of IH after kidney transplantation and create a prediction model applicable at the day of transplantation. Four factors of importance were identified, namely age at transplantation, BMI at transplantation, duration of renal replacement therapy and the “modified Israelsson wound closure technique”. The strongest and the only easily modifiable predictor was the use of “modified Israelsson”, reducing the odds of IH by 83%. Previous studies have shown superior graft survival in living donor KT compared with deceased donor kidney transplantation [17, 18], but no study thus far has compared IH incidence in relation to donor source. Notably, transplant recipients of living donor kidneys tend to be younger and with less comorbidity, which is possibly a significant confounder that could influence outcomes [19, 20].

Our final predictive model, incorporating age, BMI, duration of renal replacement therapy, and wound closure technique, had a Nagelkerke pseudo R² of 0.15. While this indicates that these factors contribute to explaining the variance in IH development, the value also suggests that other unmeasured variables likely play a role.

Sarcopenia, described by skeletal muscle index and fatty muscle index, was not a significant factor for IH in the multivariate logistic regression analysis. This is in line with a previous study performed on midline incisions by van Rooijen [10].

In this study, the Penn Hernia Risk Calculator [7] did not predict IH after KT. The model may be too general to be fully applicable to patients with kidney failure. It also omits the wound closure technique, which, according to our analysis, seems to be the strongest risk factor of IH in patients with kidney failure. It seems that, especially in the kidney transplant setting, a more specific prediction model for IH development is of great clinical value.

Our study has several limitations. First, the primary outcome in our study was symptomatic IH, according to data obtained retrospectively from medical records. While symptomatic IH is a clinically relevant endpoint and frequently used in hernia literature, relying on reported symptoms might underestimate the true incidence, as asymptomatic or mildly symptomatic hernias could be overlooked. This could mean that the actual number of IH cases is higher, and consequently, the protective effect of the “modified Israelsson technique” even more pronounced than suggested by our current estimates. Future prospective studies using standardized imaging protocols for all patients would be beneficial to capture the full spectrum of IH.

Second, a significant limitation is that the modified Israelsson technique was consistently performed by only one surgeon. While the total number of surgeries utilizing this technique was substantial (97 cases, 14% of the analysed procedures), and two other surgeons performed a higher volume of transplants (24% and 17% of analysed procedures respectively) without a corresponding reduction in hernia rates using other techniques, this single-surgeon concentration of the modified Israelsson method potentially introduces a confounder, i.e. an unmeasured surgeon-specific expertise or subtle patient selection bias that we could not control for in this retrospective analysis. Future studies are needed to evaluate the generalizability of the technique. Information about wound closure technique was retrieved from the surgery reports, and in cases of ambiguity, a questionnaire was sent to the involved surgeons; however, if the technique could not be definitively determined, these cases were not classified as “modified Israelsson,” potentially defaulting to the standard closure group if details were insufficient.”

Furthermore, regarding the sarcopenia analysis, preoperative CT scans were available for only 40% of the study population and were performed within a 36-month window prior to transplantation, after progression to CKD-5. This timeframe and limited availability could impact the accuracy of sarcopenia assessment at the time of transplantation and introduce selection bias.

In summary, the wound closure technique appears to play a critical role in the formation of IH following KT. Other risk factors for IH, while potentially contributing, seem to be of lesser significance and are often more challenging to address. To further minimize IH risk, efforts to prevent postoperative wound infections and improve transplant function should be prioritized. Implementing the “modified Israelsson wound closure technique” is strongly recommended, as it is a safe, simple, and most probably a very cost-effective approach to reduce the risk of symptomatic IH after KT.
